# Association between serum level of urate and subclinical atherosclerosis: results from the SCAPIS Pilot

**DOI:** 10.1186/s13075-020-2119-0

**Published:** 2020-02-22

**Authors:** Panagiota Drivelegka, Helena Forsblad-d’Elia, Oskar Angerås, Göran Bergström, Caroline Schmidt, Lennart T. H. Jacobsson, Mats Dehlin

**Affiliations:** 1 Department of Rheumatology and Inflammation Research, Institute of Medicine, The Sahlgrenska Academy at University of Gothenburg, Sahlgrenska University Hospital, Grona Straket 12, 413 45 Gothenburg, Sweden; 2 Department of Public Health and Clinical Medicine, Rheumatology, Umeå University, Umeå, Sweden; 3 Department of Cardiology, Sahlgrenska University Hospital, Gothenburg, Sweden; 4 Department of Molecular and Clinical Medicine, The Sahlgrenska Academy, University of Gothenburg, Gothenburg, Sweden; 5 Department of Clinical Physiology, Region Västra Götaland, Sahlgrenska University Hospital, Gothenburg, Sweden; 6 Wallenberg Laboratory for Cardiovascular Research, Institution for Medicine, Department of Molecular and Clinical Medicine, The Sahlgrenska Academy at Gothenburg University, Gothenburg, Sweden

**Keywords:** Serum urate, SCAPIS, Subclinical atherosclerosis, Coronary artery calcification, Intima-media thickness, Carotid plaque, Cardiovascular disease

## Abstract

**Background:**

Hyperuricemia is closely associated with cardiovascular disease (CVD). However, it has not been definitively established whether this association is independent of traditional cardiovascular risk factors (CVRFs) and whether it is gender-dependent. The aim of this study was to investigate in a population-based cohort (age range, 50–64 years) stratified by sex the association between the serum urate (SU) concentration and subclinical atherosclerosis, as reflected in the coronary artery calcification (CAC) score, common carotid intima-media thickness (CIMT), and carotid plaque score.

**Methods:**

The study involved participants in the Swedish CArdioPulmonary bioImage Study (SCAPIS) Pilot cohort (*N* = 1040; 48.8% males). This pilot cohort is part of the large population-based SCAPIS with 30,000 participants in the age range of 50–64 years, aimed at improving risk prediction for CVD. Subjects with a self-reported previous history of CVD (*N* = 68) or gout (*N* = 3) were excluded. The CAC score was assessed with the Agatston method using computed tomography. CIMT and carotid plaques were quantified by ultrasound. The associations between the SU quartiles and different levels of CAC, CIMT, and carotid plaques were assessed by multivariable logistic regression.

**Results:**

Age, BMI, education level, smoking, physical activity, hs-CRP, hypertension, and dyslipidemia showed no differences between males and females, while CAC (score > 0) and diabetes were both twice as common in men than in women (58% vs 26% and 8% vs 4%, respectively). Higher SU quartiles were in both sexes associated with BMI, hs-CRP, and the prevalence of hypertension, and in women, they were also associated with the prevalence of dyslipidemia. The three upper quartiles of SU (>308μmol/L) were linked to higher CAC scores in men, when adjusting for CVRFs, but not in women. CIMT and carotid plaques showed no correlation to SU in either sex.

**Conclusions:**

Higher levels of SU are associated with the presence of CAC in men but not in women, whereas SU is not associated with CIMT or carotid plaques in either men or women. This implies that the biological effects of SU differ in men and women or that SU has varying effects on different vascular beds or during the different stages of the atherosclerotic process.

## Background

### Urate levels and cardiovascular risk

Hyperuricemia is closely associated with cardiovascular disease (CVD), although it has not been definitively established whether this is due to covariation with the traditional cardiovascular risk factors (CVRFs) or a causative role of its own [1]. High levels of serum urate (SU) have been identified as an independent risk factor for hypertension (HT) [2], an association that is also supported by experimental studies [3]. In addition, higher levels of SU are also strongly linked to metabolic syndrome (MS), hyperlipidemia, reduced kidney function, higher BMI, and more advanced age [1]. Furthermore, higher levels of SU are seen in men than in women [1].

### Prediction of cardiovascular risk

Accurate assessment of atherosclerosis in its subclinical phase has important implications for early intervention and management. The European Society of Cardiology [4] and the American Heart Association [5] both recommend coronary artery calcification (CAC) score as a complement to risk prediction for individuals who have no known CVD, but who carry an intermediate risk of myocardial infarction (MI) or cardiovascular (CV) death. The detection of CAC using computed tomography (CT) scanning is highly predictive of the presence of histopathologic atherosclerosis [6], and the extent of calcification correlates strongly with the presence of carotid plaques [7]. Ultrasound of the carotid artery that identifies increases in the carotid intima-media thickness (CIMT) and carotid plaques has been shown to predict increased risk of CVD [8–10].

Associations between the SU concentration and subclinical atherosclerosis, as reflected by CAC score, CIMT, and carotid plaque score, have previously been studied but have produced conflicting findings. Increased SU levels were shown to be associated with greater risk for CAC development and progression in some previous studies [11–21], although not in others [22–24]. Regarding the relationship between SU and CIMT, an independent association has been reported in some studies [17, 25–31], whereas other studies have showed no such correlation [32, 33]. In addition, studies that have examined the relationship between increased SU levels and carotid plaque scores have demonstrated an independent association [34, 35].

The Swedish CArdioPulmonary bioImage Study (SCAPIS) is a large, population-based study that has been initiated in Sweden with the aims of improving risk prediction for CVD and optimizing the ability to study disease mechanisms. SCAPIS has recruited 30,000 subjects in the age range of 50–64 years, randomly selected from the Swedish population register. The study design is described in detail elsewhere [36]. The SCAPIS Pilot, used in the present study, is the first part of SCAPIS; it was conducted at Sahlgrenska University Hospital in Gothenburg, Sweden, in 2012 to test the design of the larger study.

The aim of the present study was to investigate the associations between SU levels and three markers of subclinical atherosclerosis, i.e., CAC, CIMT, and carotid plaques, with stratification according to gender.

## Methods

This was a population-based, cross-sectional analysis to examine the possible association between SU and subclinical atherosclerosis in patients who participated in the SCAPIS Pilot.

All the participants gave written informed consent on their first visit. Ethical approval for this study was granted by the Ethical Review Board of Gothenburg and the Ethical Review Board of Umeå, Sweden (permit no. 673-16).

### Study population

This study was conducted on individuals who participated in the SCAPIS Pilot. In this pilot study, a randomly selected population sample from the Western Sweden Health Care Region (WSHCR), consisting of 2243 adults in the age range of 50–64 years, was selected from the census register. Of the selected persons, 1111 subjects agreed to participate (50% participation rate). A particularly low participation rate was seen for individuals who were born outside Europe, were living alone in an area with low socioeconomic status, had a low level of education, found themselves outside the labor market, and had a low income [37]. We excluded subjects who had gout and/or urate-lowering treatment, based on free-text self-reported data under “other diseases/medication” (*N* = 3), as well as those with a previous history of CVD (*N* = 68), defined as either answering positively to the question: “Have you ever been told by a physician that you had a myocardial infarction (MI), stroke or presence of coronary stent?” or the detection of a coronary stent in a CT-image. Finally, 1040 participants were included; all of them were 50 to 64 years old at examination.

The study subjects originated in equal proportions from residential areas with high and low socioeconomic status, respectively. All the study subjects responded to a questionnaire regarding smoking habits (never, occasional, previous or active), level of education (0–9 years, 10–12 years, or > 12 years), physical activity, and self-reported health and medication. The diagnoses of hypertension, diabetes, and dyslipidemia were based on self-reported data and were defined as answering positively to the question: “Have you ever been told by a physician that you have hypertension?”, “Have you ever been told by a physician that you have diabetes?”, and “Have you ever been told by a physician that you have dyslipidemia”, respectively. Physical activity was classified according to four levels: 1, less than 2 h per week; 2, moderate, for at least 2 h per week to induce a sweat; 3, moderate and regularly, at least one or two times per week and for at least 30 min; and 4, regularly, at least three times per week and for at least 30 min. The levels of creatinine, high-sensitivity CRP (hs-CRP), and SU were measured for all subjects at study entry. Kidney function was categorized based on estimated glomerular filtration rate (eGFR), of > 90 mL/min, 60–90 mL/min, and < 60mL/min, as calculated using the Chronic Kidney Disease Epidemiology Collaboration (CKD-EPI) equation [38].

### Measurements of subclinical atherosclerosis

#### Coronary artery calcification (CAC) score

The calcium content of each coronary artery was measured with CT and summed to produce a total coronary artery calcification (CAC) score according to the Agatston method [7, 36]. Participants were classified into three groups according to CAC score of 0, 1–100, and > 100, respectively. A total CAC score > 0 was considered to indicate positivity for the presence of CAC.

#### Common carotid intima-media thickness (CIMT)

Atherosclerosis of the carotid arteries was assessed using a standardized protocol with a Siemens Acuson S2000 ultrasound scanner equipped with a 9L4 linear transducer [36]. The two-dimensional (2D) greyscale ultrasound image was analyzed to determine the intima-media thickness. CIMT was calculated as the mean of the intima-media thicknesses of the left and right common carotid arteries, and it was categorized as follows: < 25th percentile, 25th–75th percentile, and > 75th percentile. The CIMT > 75th percentile was considered to be positive.

#### Carotid plaques

The presence of plaque was defined according to the Mannheim Consensus [39].

A plaque score was calculated by summing the largest plaque size in each of right carotid artery, right bulb, right internal carotid artery, left carotid artery, left bulb, and left internal carotid artery (range, 0–24). The largest plaque in each artery was scored as follows: 2, small plaque; 3, moderate plaque; and 4, plaque affecting the blood flow velocity (maximum score, 24).

Participants were categorized into three groups, according to plaque scores of 0, 1–2, and > 2, respectively. Any sign of carotid plaque on the ultrasound examination (plaque score > 0) was considered to be positive for the presence of carotid plaque.

### Statistical analysis

Baseline characteristics are expressed as absolute counts and proportions for categorical variables and as means ± standard deviations (SD) for continuous variables.

Univariable and multivariable logistic regression models were used to assess the associations of SU with CAC, CIMT, and carotid plaques after adjustment for age, smoking, body mass index (BMI), eGFR, diabetes, dyslipidemia, hypertension, hs-CRP, physical activity level, and education level. In the logistic regression models, CAC score > 0, CIMT > 75th percentile, and plaque score > 0 were considered to represent pathologic findings and used as the cutoff values for the CAC score, CIMT, and carotid plaque score, respectively. The level of significance was set at *p* < 0.05. All analyses were performed using the SAS ver. 9.3 software (SAS Institute Inc., Cary, NC, USA).

## Results

### Characteristics of the participants and associations with the level of serum urate

Among the participants, 508 (48.8%) were men (Table 1). The mean ages were 57.7 ± 4.4 years for men and 57.5 ± 4.3 for women (Table 1). There were no significant differences in age, BMI, hs-CRP, smoking status, hypertension, and dyslipidemia between the sexes, whereas diabetes was twice as common in men (8% vs 4%) (Table 1). The majority of the men (62%) had an eGFR of 60–90 mL/min, whereas the majority of the women (88%) had an eGFR > 90 mL/min (Table 1).

**Table 1:** Characteristics of the subjects stratified by gender and serum urate quartiles

	Men (*N* = 508)					Women (*N* = 532)				
	Total, (*N* = 508)	1st quartile 31–307 μmol/L (*N* = 124)	2nd quartile 308–346 μmol/L (*N* = 132)	3rd quartile 347–391 μmol/L (*N* = 127)	4th quartile 392–584 μmol/L (*N* = 125)	Total (*N* = 532)	1st quartile 143–229 μmol/L (*N* = 132)	2nd quartile 230–262 μmol/L (*N* = 131)	3rd quartile 263–304 μmol/L (*N* = 135)	4th quartile 305–702 μmol/L (*N* = 134)
Age in years, mean (SD)	57.7 (4.4)	57.8 (4.5)	57.8 (4.4)	56.9 (4.6)	58.2 (4.1)	57.5 (4.3)	56.8 (4.0)	57.6 (4.4)	57.5 (4.2)	58.3 (4.6)
BMI, mean (SD)	27.7 (4.1)	26.1 (3.2)	27.3 (3.6)	28.3 (4.0)	29.2 (4.7)	26.8 (4.9)	24.5 (3.6)	25.2 (3.4)	26.9 (4.5)	30.4 (5.7)
Smoking, *N* (%)										
Never	215 (42)	49 (40)	66 (50)	53 (42)	47 (38)	237 (45)	51 (39)	60 (46)	66 (49)	60 (45)
Occasional	12 (2)	3 (2)	2 (2)	5 (4)	2 (2)	23 (4)	10 (8)	5 (4)	4 (3)	4 (3)
Previous	206 (41)	48 (39)	49 (37)	51 (41)	58 (46)	191 (36)	47 (36)	46 (35)	51 (38)	47 (35)
Active	74 (15)	24 (19)	16 (12)	16 (13)	18 (14)	79 (15)	23 (18)	20 (15)	14 (10)	22 (17)
Physical activity, *N* (%)										
Less than 2 h per week	89 (18)	22 (18)	21 (16)	24 (20)	22 (18)	75 (14)	14 (11)	9 (7)	20 (15)	32 (24)
Moderate, for at least 2 h per week to induce a sweat	205 (41)	50 (41)	48 (36)	51 (42)	56 (45)	242 (46)	65 (50)	57 (44)	58 (43)	62 (47)
Moderate and regularly, at least one or two times per week and for at least 30 min	140 (28)	29 (24)	45 (34)	32 (26)	34 (27)	120 (23)	29 (22)	34 (26)	29 (22)	28 (21)
Regularly, at least three times per week and for at least 30 min	68 (14)	21 (17)	18 (14)	16 (13)	13 (10)	91 (17)	23 (18)	31 (24)	27 (20)	10 (8)
eGFR, *N* (%)										
eGFR, > 90 mL/min	180 (35)	64 (52)	46 (34)	40 (32)	30 (24)	467 (88)	125 (95)	118 (90)	116 (86)	108 (81)
eGFR, 60–90 mL/min	316 (62)	60 (48)	85 (63)	82 (66)	89 (71)	60 (11)	5 (4)	13 (10)	17 (13)	25 (19)
eGFR, < 60 mL/min	12 (2)	0 (0)	3 (2)	3 (2)	6 (5)	5 (1)	2 (2)	0 (0)	2 (2)	1 (1)
SU, μmol/L, mean (SD)	353 (67)	273 (34)	327 (12)	370 (12)	441 (40)	270 (63)	200 (22)	245 (10)	281 (12)	353 (52)
hs-CRP, mean (SD)	2.2 (3.4)	1.7 (2.8)	1.6 (1.6)	2.5 (2.9)	3.1 (5.2)	2.4 (3.8)	1.4 (1.4)	2.2 (5.7)	2.1 (2.7)	3.9 (3.7)
Hypertension, *N* (%)	160 (32)	26 (21)	38 (29)	47 (40)	49 (40)	178 (34)	30 (23)	35 (28)	47 (36)	66 (50)
Diabetes, *N* (%)	39 (8)	10 (8)	11 (8)	10 (8)	8 (7)	21(4)	6 (5)	3 (2)	3 (2)	9 (7)
Dyslipidemia, *N* (%)	144 (30)	30 (25)	38 (30)	35 (30)	41 (35)	121 (24)	28 (22)	29 (23)	22 (17)	42 (34)
CAC+, *N* (%)	293 (58)	59 (48)	80 (60)	73 (58)	81 (65)	137 (26)	31 (24)	31 (24)	33 (25)	42 (31)
CIMT+, *N* (%)*	106 (24)	24 (22)	34 (30)	20 (19)	28 (27)	117 (25)	24 (21)	29 (24)	31 (26)	33 (29)
Plaque+, *N* (%)*	308 (61)	68 (55)	88 (66)	72 (58)	80 (64)	268 (51)	68 (52)	60 (47)	71 (53)	69 (52)

CAC+: total CAC score > 0

CIMT+: CIMT > 75th percentile

Plaque+: plaque score > 0

*Because of missing values, the total number of subjects in each urate quartile is:

For CIMT and men: 1st quartile *N* = 111, 2nd quartile *N* = 115, 3rd quartile *N* = 106, and 4th quartile *N* = 104

For CIMT and women: 1st quartile *N* = 117, 2nd quartile *N* = 123, 3rd quartile *N* = 120, and 4th quartile *N* = 115

For plaque score and men: 1st quartile *N* = 124, 2nd quartile *N* = 133, 3rd quartile *N* = 125, and 4th quartile *N* = 125

For plaque score and women: 1st quartile *N* = 131, 2nd quartile *N* = 128, 3rd quartile *N* = 134, and 4th quartile *N* = 133

All the participants were stratified by gender and four quartiles according to their SU level (Table 1). For men, these quartiles were as follows: first, 31–307 μmol/L; second, 308–346 μmol/L; third, 347–391 μmol/L; and fourth, 392–584 μmol/L. In women, the corresponding quartiles were as follows: first, 143–229 μmol/L; second, 230–262 μmol/L; third, 263–304 μmol/L; and fourth, 305–702 μmol/L.

For both sexes, higher SU quartile values were associated with BMI (*p* < 0.0001 in both men and women), hs-CRP (*p* = 0.007 in men and *p* < 0.0001 in women), and the prevalence of hypertension (*p* = 0.003 in men and *p* < 0.0001 in women). Higher SU quartile values were also significantly associated with dyslipidemia (*p* = 0.019) and lower socioeconomic status (*p* = 0.001) in women, but not in men (*p* = 0.394 for dyslipidemia and *p* = 0.879 for socioeconomic status) (Table 1, Additional file 1: Table S1). The proportions of both men and women with eGFR > 90 mL/min were lower in those with higher SU quartile values, whereas the proportions with moderately reduced kidney function (i.e., eGFR in the range of 60–90 mL/min) were higher. The highest level of physical activity was less frequently in those with higher SU quartile values in both sexes, whereas no associations were detected between the SU quartiles and education level, smoking status, and prevalence of diabetes in either of the sexes (Table 1).

### CAC

**Fig. 1:**
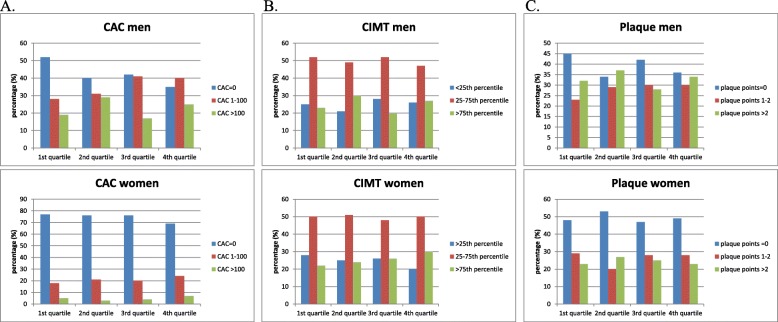
**a** Associations between SU concentrations and different CAC scores in men and women. **b** Associations between SU concentrations and different percentiles of CIMT in men and women. **c** Associations between SU concentrations and different plaque scores in the carotid arteries of men and women

CAC was determined in all the subjects. Among the men, 58% were CAC-positive (CAC > 0), whereas among the women, only 26% were CAC-positive. Figure 1 shows the associations between the SU quartiles and different levels of CAC scores. The three upper quartiles of SU (> 308 μmol/L) were statistically associated with the presence of CAC in men (*p* < 0.05), but not in women, when adjustments were made for age, smoking, BMI, diabetes, dyslipidemia, hypertension, eGFR, hs-CRP, education level, and physical activity in a multivariable logistic regression (Table 2).

**Table 2:** Odds ratios (OR) for SU quartiles and for being CAC-positive, CIMT-positive, and plaque-positive, stratified by sex. Data shown are for univariable and multivariable logistic regressions, with adjustments for age, smoking, BMI, diabetes, dyslipidemia, eGFR, hypertension, hs-CRP, physical activity, and education level

Urate quartiles, μmol/L	Men (*N* = 508)				Urate quartiles, μmol/L	Women (*N* = 532)			
	OR, univariable	*p* value	OR, multivariable	*p* value		OR, univariable	*p* value	OR, multivariable	*p* value
	CAC examined (*N* = 508)^a^ CAC+ (*N* = 293)					CAC examined (*N* = 532)^a^ CAC+ (*N* = 137)			
31–307, ref	1		1		143–229, ref	1		1	
308–346	1.6 (1.0–2.7)	0.049	2.2 (1.2–4.0)	0.008	230–262	1.0 (0.6–1.8)	0.97	0.8 (0.4–1.6)	0.5
347–391	1.6 (0.9–2.5)	0.08	1.9 (1.0–3.6)	0.04	263–304	1.1 (0.6–1.9)	0.8	1.0 (0.5–1.9)	0.9
392–584	2.0 (1.2–3.4)	0.007	2.3 (1.2–4.4)	0.01	305–702	1.5 (0.9–2.6)	0.2	1.0 (0.5–2.0)	0.96
	CIMT examined (*N* = 436)^a^ CIMT+ (*N* = 106)					CIMT examined (*N* = 475)^a^ CIMT+ (*N* = 117)			
31–307, ref	1		1		143–229, ref	1		1	
308–346	1.5 (0.8–2.7)	0.2	1.2 (0.6–2.4)	0.5	230–262	1.2 (0.6–2.2)	0.6	1.2 (0.6–2.3)	0.7
347–391	0.9 (0.5–1.7)	0.7	0.7 (0.3–1.4)	0.3	263–304	1.3 (0.7–2.4)	0.4	1.0 (0.5–2.1)	0.9
392–584	1.3 (0.7–2.5)	0.4	0.9 (0.4–1.8)	0.7	305–702	1.6 (0.9–2.9)	0.1	1.0 (0.5–2.2)	0.99
	Carotid plaque examined (*N* = 507)^a^ Plaque+ (*N* = 308)					Carotid plaque examined (*N* = 526)^a^ Plaque+ (*N* = 268)			
31–307, ref	1		1		143–229, ref	1		1	
308–346	1.6 (0.95–2.6)	0.08	1.8 (1.0–3.2)	0.03	230–262	0.8 (0.5–1.3)	0.4	0.9 (0.5–1.6)	0.7
347–391	1.2 (0.7–1.9)	0.6	1.1 (0.6–2.0)	0.7	263–304	1.1 (0.7–1.7)	0.8	1.3 (0.7–2.2)	0.4
392–584	1.5 (0.9–2.4)	0.1	1.6 (0.9–2.9)	0.2	305–702	0,99 (0.6–1.6)	0.95	1.1 (0.6–2.1)	0.7

^a^The total number of subjects, where CAC score, CIMT, and plaque score respectively, were calculated

CAC+: total CAC score > 0

CIMT+: CIMT > 75th percentile

Plaque+: plaque score > 0

### CIMT

CIMT was determined in 436 men and 475 women. Figure 1 shows the associations between the SU quartiles and the different levels of CIMT. Comparing the SU quartiles with CIMT, no significant association was found in either the men or women after adjustments were made for age, smoking, BMI, diabetes, dyslipidemia, hypertension, eGFR, hs-CRP, education level, and physical activity in a multivariable logistic regression (Table 2).

### Carotid plaque

Carotid plaque was determined in 507 men and 526 women. Figure 1 shows the associations between the SU quartiles and the different levels of plaque score. Among the male participants, 61% had signs of carotid plaques, whereas only 51% of the women had signs of carotid plaques. When comparing the SU quartiles with the presence of carotid plaque, no significant association was found in either the men or women after adjustments were made for age, smoking, BMI, diabetes, dyslipidemia, hypertension, eGFR, hs-CRP, education level, and physical activity in a multivariable logistic regression (Table 2).

## Discussion

In this population-based and cross-sectional pilot study of the SCAPIS cohort, we show that higher levels of SU are significantly associated with the presence of CAC in men but not in women, when adjusting for traditional CVRFs. In contrast, the levels of SU show no association with common CIMT or carotid plaques in either men or women.

### SU and CAC

A few prospective and several cross-sectional studies have examined the association between SU level and CAC. All three prospective studies [12, 14, 15] have shown that a higher SU level predicts progression of CAC over time. In particular, Rodrigues et al. [12], in a 6-year follow-up study of 443 individuals (with type 1 diabetes, but without diagnosed coronary artery disease) and 526 control subjects, found that the baseline level of SU (mean, 5.6 mg/dL) predicted CAC progression in those persons with normal renal function. The CAC-progressors were older and had a higher CAC at baseline than the non-progressors. The levels of SU were significantly higher in the CAC-progressors (mean, 5.6 mg/dL; range, 4.9–6.5 mg/dL) than in non-progressors (mean, 5.1 mg/dL; range, 4.4–5.9 mg/dL), *p* < 0.0001). Bjornstad et al. [14] studied 652 adults (46% men; mean age, 37 ± 9 years for men and 36 ± 9 years for women) with type 1 diabetes at baseline and 6 years later and found that the level of SU (concentrations at baseline, 5.6 ± 1.0 mg/dL in men and 4.6 ± 1.0 mg/dL in women) independently predicted the progression of CAC. Calvo et al. [15] studied 202 white and 166 Filipino postmenopausal women without known CVD (mean SU concentration at baseline, 262.2 μmol/L) (follow-up at 4.6 years) and found that SU was independently associated with increased CAC severity in Filipino women and with CAC progression in both groups. Many cross-sectional studies have been performed, two of which included only men. Santos et al. [16] studied 378 Brazilian men without known CVD (mean age, 48 years) and Zhang et al. [17] studied 3010 healthy Korean men (57% of whom were aged > 50 years). Both studies showed that SU was independently associated with the presence of CAC (CAC > 0). Among the remaining cross-sectional studies, Kim et al. [18] studied 4188 Korean men and women with mean age of 53 years and mean SU concentration of 5.4 mg/dL and showed a positive association between SU and CAC in older males with BMI < 25 and without diabetes, hypertension, smoking, or renal dysfunction. The study conducted by Krishnan et al. [19] included 2498 healthy individuals (48% men; mean age, 40 years) and showed that SU (mean concentrations, 5.8 mg/dL in men and 4.0 mg/dL in women) independently correlated to CAC levels, although 90% of the cohort was CAC-negative. The study by Grossman et al. [20] included 663 individuals from the CARDIA trial (85% men; mean age, 55.5 years) without known CVD and showed an independent correlation between SU (mean concentration, 5.5 mg/dL) and CAC. Atar et al. [21] also found an independent association between SU and CAC in a study of 442 individuals from Turkey (77% male; mean age, 49 years; 54% CAC-negative).

There have also been cross-sectional studies that did not show any association between SU and CAC levels. Coutinho et al. [22] in a study of 1107 sibships (41% men; mean age, 58.1 years) with at least two members who had hypertension diagnosed before the age of 60 years showing no association between SU (mean concentration, 6.0 mg/dL) and the presence or severity of CAC in men or women when adjustment was made for traditional CVRFs. Malik et al. [23] in a cohort of 208 Brazilian octogenarians (78.9% women) showed no significant difference with respect to the presence of CAC across increasing SU tertiles in a multivariable analysis. Neogi et al. [24] in a multicenter study of 2412 white participants recruited from American, population-based cohorts showed that SU (mean concentrations, 6.1 mg/dL in men and 4.7 mg/dL in women) was not associated with CAC independently of other CVRFs.

Our results showing a non-linear association in men and no association in women between SU and CAC are in line with the associations, demonstrated in most other studies. The discrepancies between the studies could have several explanations, including publication bias against negative results and heterogeneity with regard to sample sizes and characteristics (age, sex, ethnicity, degree of comorbidities), as well as adjustments for confounders in the study populations.

### SU and CIMT

Several cross-sectional studies have examined the association between SU and CIMT. In agreement with the results of the present study, Bae et al. [32] found no differences in CIMT between non-hyperuricemic and hyperuricemic (mean SU levels, 5.7 mg/dL in men and 4.2 mg/dL in women) groups in either men or women in a Korean multi-rural communities cohort of 5568 healthy participants aged > 40 years (39% men; mean ages, 61.5 years for men and 59.5 years for women), which aimed at identifying risk factors for CVD. Iribarren et al. [33] examined the participants in the ARIC study (6522 women, 74% white; and 4966 men, 79% white; age range at baseline, 45–64 years) and found no association between SU (mean concentration, 5.9 mg/dL) and CIMT independent of other CVRFs in either males or females. The Young Finns Study [40] of 1985 young adults (46.5% men), in the age range of 30–45 years, also reported no evidence of uric acid involvement (mean concentrations, 330.7 μmol/L in men and 241.3 μmol/L in women) in carotid atherosclerosis, although they showed that serum uric acid was linked to cardiovascular risk markers, most notably BMI. Finally, in a cohort study of 359 consecutive patients who were undergoing coronary angiography and carotid ultrasound, De Luca et al. [41] found no association between SU and CIMT.

In contrast, Kawamoto et al. [29] conducted a study of 1128 inpatients at a medical department (44% men; mean ages, 68 years for men and 72 years for women) and found that the second (4.3–5.2 mg/dL), third (5.3–6.3 mg/dL), and fourth (6.4–10.0 mg/dL) quartiles of the SU concentration were independently associated with carotid atherosclerosis in men without metabolic syndrome, although not in women. Takayama et al. [31] in a Japanese study of an elderly population (1579 participants; 42% men; mean ages, 78 years for men and 79 years for women) found that CIMT was significantly increased in the third (5.6–6.8 mg/dL) and fourth (6.9–16.1 mg/dL) SU quartiles in men without metabolic syndrome and in the fourth quartile (6.1–15.4 mg/dL) in women without metabolic syndrome, independently of other risk factors. Both Moltacini et al. [25] in a study of 234 postmenopausal women without a history of CVD (mean age, 56.4 years; mean SU concentration, 4.2 mg/dL) and Zhang et al. [17] in a large study of 3010 male Koreans found that SU was associated with CIMT independently of other risk factors. A significant association, after adjustment for other risk factors, was also found in a study of 619 healthy individuals (40% men; mean age, 53 years; mean SU concentration, 4.8 mg/dL) by Cicero et al. [27], as well as in a study of 530 individuals (60.6% men; mean age, 58.9 years; mean SU concentration, 347 μmol/L) at increased cardiovascular risk [26].

Our results are in line with some previous studies, but differ from others, where often the study population either had a poorer health profile or was older.

### SU and carotid plaques

A few cross-sectional studies have examined the association between SU and carotid plaques. In contrast to our findings, the study performed by Neogi et al. [35] with 4866 Americans (46% male; mean age, 52 years) without risk factors related to CVD and hyperuricemia showed an independent association between SU and carotid plaques in men (mean SU concentration, 6.2 mg/dL) but not in women (mean SU concentration, 4.8 mg/dL). In addition, Ishizaka et al. [34] showed higher prevalences of carotid plaques in the second (5.4–6.1 mg/dL), third (6.2–7.0 mg/dL), and fourth (7.1–11.0 mg/dL) quartiles of the SU concentrations in a study of 8144 Japanese individuals without metabolic syndrome (67% male; mean age, 56 years), whereas they found no such association in either men with metabolic syndrome or in women overall.

It is not clear why these results contrast with our findings, although differences between the study populations, with regard to SU or ethnicity, are possible explanations.

### Sex differences

Regarding the differences in the association between SU and CAC between the sexes noted in the present study, a possible explanation is the inclusion of a higher proportion of CAC-positive men (58%) than women (26%). Another possible explanation is that men develop atherosclerosis and CVD much earlier in life than women [42]. Calcification may be regarded as a later stage of atherosclerosis in the arteries. In the atherosclerotic process, fatty deposits initially appear in the inner layers of the arteries, and small cholesterol crystals are deposited in the intima and its underlying smooth muscle. The plaques grow concomitant with the proliferation of fibrous tissues and the surrounding smooth muscle and, finally, connective tissue production by fibroblasts and the deposition of calcium in the lesion result in sclerosis and stiffening of the arteries [43]. Furthermore, men are exposed to higher levels of SU throughout life, which may explain the more potent effect of SU on calcification in men, as compared to women, in whom the levels of SU increase substantially first after menopause [44].

The gender difference in relation to the occurrence of pathologic vascular findings was also reflected in the higher frequency of carotid plaques in men compared to women, at 61% vs 51%, respectively (*p* = 0.0004). However, SU did not correlate to CIMT or carotid plaques. This implies that SU exerts different effects on carotid arteries than on coronary arteries or that SU has specific effects on the calcification process that are not related to the initiation or early signs of atherosclerosis.

### Strengths and limitations

Some possible limitations should be acknowledged. First, there was a relatively low participation rate (50%) and some selection bias towards higher socioeconomic levels, which may hamper the generalizability of the outcomes. It is known from other health surveys that study participants tend to be healthier than non-participants [37, 45]. Second, the relatively low age of the participants rendered a relatively low prevalence of subclinical signs of atherosclerosis, in particular for women. Third, due to a lack of data, we could not provide information about the ethnicities of the participants, although this is likely to have had a minor impact on the results of the present study, as the overwhelming majority of the participants (88.2%) were born in Sweden or in other European countries. Fourth, the diagnosis of gout was based on free-text self-reported data, which may have resulted in lower occurrence of gout among the participants. Fifth, the cross-sectional design of the present analysis limits the possibility to study the causality of the observed associations.

The main strengths of this study are the relatively large size of the cohort and the fact that the participants were chosen from the census register, which guarantees the representativeness of the study population and the generalizability of the results.

## Conclusions

In summary, in the present study, we show that higher levels of SU are significantly associated with the presence of CAC in men in a nonlinear fashion, but not in women. We also show that SU level is not associated with CIMT or carotid plaques in either men or women. These results imply different biological effects of SU in men and women or reflect different vascular beds or stages of the atherosclerotic process.

## Supplementary information

**Additional file 1:** **Table S1.** Education level and socioeconomic status of the subjects stratified by gender and serum urate quartiles.

## Data Availability

The datasets used and/or analyzed during the current study are available from the corresponding author upon reasonable request.

## Abbreviations

BMI: Body mass index
CAC: Coronary artery calcification
CIMT: Carotid intima-media thickness
CT: Computed tomography
CV: Cardiovascular
CVD: Cardiovascular disease
CVRFs: Cardiovascular risk factors
eGFR: Estimated glomerular filtration rate
hs-CRP: High-sensitivity C-reactive protein
HT: Hypertension
MI: Myocardial infarction
MS: Metabolic syndrome
SCAPIS: Swedish CArdioPulmonary bioImage Study
SD: Standard deviation
SU: Serum urate
WSHCR: Western Swedish Health Care Region
