# Supplementary material for: Association between serum level of urate and subclinical atherosclerosis: results from the SCAPIS Pilot
Source: Arthritis Res Ther. 2020 Feb 22;22:37. doi: 10.1186/s13075-020-2119-0 (PMC7036243; doi:10.1186/s13075-020-2119-0)
Supplement: Supplementary file 1 — Additional file 1: Table S1. Education level and socioeconomic status of the subjects stratified by gender and serum urate quartiles. [file 13075_2020_2119_MOESM1_ESM.docx]

|  | **Men,**  **(N=508)** | | | | | **Women,**  **(N=532)** | | | | |
| --- | --- | --- | --- | --- | --- | --- | --- | --- | --- | --- |
|  | **Total, (N=508)** | **1^st^Quartile**  **31-307 µmol/L, (N=124)** | **2^nd^Quartile 308-346 µmol/L, (N=132)** | **3^rd^Quartile 347-391 µmol/L, (N=127)** | **4^th^Quartile 392-584 µmol/L, (N=125)** | **Total, (N=532)** | **1^st^Quartile**  **143-229 µmol/L, (N=132)** | **2^nd^Quartile 230-262 µmol/L, (N=131)** | **3^rd^Quartile 263-304 µmol/L, (N=135)** | **4^th^Quartile 305-702 µmol/L, (N=134)** |
|  |  | | | | | | | | | |
| **Education level,**  **N (%)** |  |  |  |  |  |  |  |  |  |  |
| *0-9 years* | 107 (21) | 29 (24) | 26 (20) | 28 (22) | 24 (19) | 90 (17) | 23 (17) | 17 (13) | 20 (15) | 30 (22) |
| *10-12 years* | 231 (45) | 60 (48) | 57 (43) | 49 (39) | 65 (52) | 224 (42) | 59 (45) | 51 (39) | 51 (38) | 63 (47) |
| *>12 years* | 170 (33) | 35 (28) | 49 (37) | 50 (39) | 36 (29) | 218 (41) | 50 (38) | 63 (48) | 64 (47) | 41 (31) |
| **Socioeconomic status of residence area, High,**  **N (%)*** | 260 (51) | 61(49) | 70 (53) | 67 (53) | 62 (50) | 275 (52) | 76 (58) | 74 (56) | 76 (56) | 49 (37) |

* p-value = 0.879 for men and p-value = 0.001 for women, when comparing socioeconomic status by quartiles of SU.

Table S1: Education level and socioeconomic status of the subjects stratified by gender and serum urate quartiles.
